# Comorbid Medical Conditions as Predictors of Overall Survival in Glioblastoma Patients

**DOI:** 10.1038/s41598-019-56574-w

**Published:** 2019-12-27

**Authors:** Matthew T. Carr, Camille J. Hochheimer, Andrew K. Rock, Alper Dincer, Lakshmi Ravindra, Fan Lily Zhang, Charles F. Opalak, Nora Poulos, Adam P. Sima, William C. Broaddus

**Affiliations:** 1 Department of Neurosurgery, Virginia Commonwealth University, Richmond, VA USA; 2 Department of Biostatistics, Virginia Commonwealth University, Richmond, VA USA

**Keywords:** Prognosis, Surgical oncology, CNS cancer

## Abstract

Glioblastoma (GBM) is an aggressive central nervous system tumor with a poor prognosis. This study was conducted to determine any comorbid medical conditions that are associated with survival in GBM. Data were collected from medical records of all patients who presented to VCU Medical Center with GBM between January 2005 and February 2015. Patients who underwent surgery/biopsy were considered for inclusion. Cox proportional hazards regression modeling was performed to assess the relationship between survival and sex, race, and comorbid medical conditions. 163 patients met inclusion criteria. Comorbidities associated with survival on individual-characteristic analysis included: history of asthma (Hazard Ratio [HR]: 2.63; 95% Confidence Interval [CI]: 1.24–5.58; p = 0.01), hypercholesterolemia (HR: 1.95; 95% CI: 1.09–3.50; p = 0.02), and incontinence (HR: 2.29; 95% CI: 0.95–5.57; p = 0.07). History of asthma (HR: 2.22; 95% CI: 1.02–4.83; p = 0.04) and hypercholesterolemia (HR: 1.99; 95% CI: 1.11–3.56; p = 0.02) were associated with shorter survival on multivariable analysis. Surgical patients with GBM who had a prior history of asthma or hypercholesterolemia had significantly higher relative risk for mortality on individual-characteristic and multivariable analyses.

## Introduction

Glioblastoma (GBM) is a common and fast-growing central nervous system (CNS) tumor with a poor prognosis. GBM, classified as World Health Organization (WHO) grade IV glioma, is a primary CNS tumor^1^. It is the most prevalent glioma (57.3%) and most common primary malignant brain tumor (14.6%) with an annual incidence rate of 3.2 per 100,000 population in the United States^1,2^. Survival time from diagnosis to death is short, with the Surveillance, Epidemiology, and End Results (SEER) database showing median overall survival ranging from 12–15 months, and the Central Brain Tumor Registry of the United States (CBTRUS) listing a 5-year survival of 6.8%^2,3^.

To date, most studies on survival^4–11^ have focused on the effects of treatment modalities^12–15^ or specific patient populations such as pediatrics^16^ and geriatrics^17,18^. Limited research has been conducted on demographic factors, clinical characteristics, or medical comorbidities as predictors for overall survival^10,19^. A few predictors of survival are well-established, including: age, extent of resection (EOR), performance status, and O^6^-methylguanine-DNA-methyltransferase (MGMT) status^10,20,21^. Other pathologic and molecular tumor biomarkers, such as epidermal growth factor receptor (EGFR) amplification, aldehyde dehydrogenase 1A3 (ALDH1A3), and isocitrate dehydrogenase (IDH1/IDH2) isoforms are current foci of research and have been linked to prognosis^22–24^. Tumor location has also been linked to prognosis in GBM patients^25^.

Given the short natural history of GBM and its prevalence among malignant brain tumors, there exists a need to elucidate further prognostic factors that can improve patient outcomes. A better understanding of medical comorbidities that affect overall survival will help determine whether or not targeted medical optimization could further improve survival in patients with GBM. Therefore, the objective of this study was to investigate demographic, clinical characteristics, and pre-existing medical comorbidities as predictors of overall survival among patients with GBM.

## Methods

Retrospective data were obtained from the Virginia Commonwealth University (VCU) Brain and Spine Tumor Registry, which contains data from medical records for all patients who presented with a brain tumor between January 2005 and February 2015. Data were extracted in August of 2017. Inclusion criteria for this study were: (1) age greater than 18, (2) pathology-confirmed diagnosis of GBM, (3) documented tumor location, (4) documented EOR, and (5) validated living status at time of censorship.

The date of first clinical encounter with VCU was considered a patient’s entry into the study. Date of death was used to define event time. Patients that were either lost to follow up or still alive at the event time were censored based on the time of their last clinic visit. Survival time was calculated as the difference between entry date and event time or censorship in days. Demographic variables included age, sex, race, insurance status, marital status, alcohol use, and tobacco use. Clinical characteristics included EOR, tumor volume, and tumor location. Performance status and MGMT promoter methylation were not included, as these variables were not available for many of the patients in the study. Tumor location was determined from pre-operative radiology reports, with “multiple locations” chosen if more than one distinct location was indicated by the radiological interpretation. EOR was determined by neurosurgery post-operative notes. EOR was graded as complete or near total if these descriptors were used in the postoperative neurosurgery notes that referenced postoperative imaging, or subtotal if the postoperative note referenced any residual contrast enhancement. Comorbidities included: arthritis, asthma, previous cancer, depression or anxiety, diabetes, heart disease, high blood pressure, hypercholesterolemia, incontinence, migraines, seizures, stomach ulcer or upset stomach, stroke, and thyroid problems. Comorbidities were collected from neurosurgery outpatient clinic and inpatient notes.

A Cox proportional hazards regression was applied to assess the relationship between each of the predictors and overall survival. First, regressions were performed to determine whether or not demographic characteristics, tumor volume, or past medical conditions were individually associated with overall survival. Predictors that were at least marginally significant (p < 0.15) were entered into a final multivariable model. All analyses controlled for age, EOR, and tumor location, as these have been shown to be prognostic factors as stated above. Estimates of overall survival were reported at six, twelve, eighteen, and twenty-four months. Models were fit using the SAS statistical software version 9.4 (Cary, NC, USA) with inferences made at the 5% level. A Kaplan-Meier survival plot was created using the R statistical software version 3.5.0 with the *survival* and *survminer* packages (Vienna, Austria). The Virginia Commonwealth University Institutional Review Board approved this study, and waived the need for informed consent as all data were pre-existing in the medical record with no additional risk to patients. All patient data were collected, stored, and analyzed in a HIPAA-compliant fashion and in accordance with VCU institutional and Collaborative Institutional Training Initiative guidelines.

## Results

A total of 197 patients diagnosed with GBM presented to VCU from January 2005 to February 2015. Of those, 163 patients met criteria for inclusion. The 34 patients who did not meet inclusion criteria were excluded primarily due to missing tumor location, missing extent of resection, or missing both variables. Table 1 lists the frequency of demographic and clinical characteristics within the study sample. There were 95 (58%) male patients. The mean age at time of diagnosis was 61.7 ± 13.3 years. A total of 124 (76%) patients identified as white race. Following surgery, 61 (37%) patients had complete tumor resection, 36 (22%) had near total resection, and 22 (14%) had subtotal resection.

**Table 1:** Demographic and clinical characteristics of sample.

Characteristic	Mean (SD) or Frequency (%)
Sex (female)	68 (42%)
Age	61.7 (13.3)
Race	
*White*	124 (76%)
*Non-white*	39 (24%)
Surgery Extent of Resection	
*Biopsy only*	44 (27%)
*Subtotal resection*	22 (14%)
*Near total resection*	36 (22%)
*Complete resection*	61 (37%)
Tumor location	
*Frontal*	34 (21%)
*Temporal*	34 (21%)
*Parietal*	13 (8%)
*Occipital*	4 (3%)
*Multiple locations*	56 (34%)
*Other*	22 (14%)
Tumor volume (cc)	79.4 (60.8)
Insurance	
*Commercial*	57 (45%)
*Government*	57 (45%)
*Uninsured*	12 (10%)
Married	104 (64%)
Alcohol use	69 (50%)
Smoking status	
*Never*	73 (72%)
*Former*	13 (13%)
*Current*	15 (15%)
Living Status	
*Alive*	1 (1%)
*Deceased*	147 (90%)
*Lost to follow-up*	15 (9%)

SD = Standard deviation.

**Figure 1:**
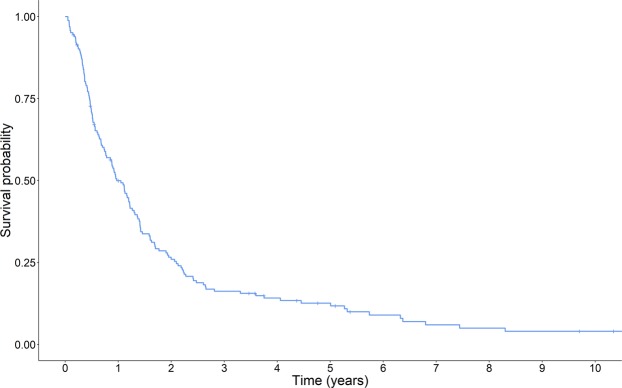
Kaplan-Meier survival plot of patients.

At the time of data collection, 147 (90%) patients were deceased, 15 (9%) patients were lost to follow-up, and 1 (1%) patient was alive. The overall probability of survival in our sample was 71% at six months, 50% at twelve months, 34% at eighteen months, and 26% at twenty-four months. Figure 1 shows a Kaplan-Meier survival curve of the overall sample.

Table 2 demonstrates the frequency of pre-existing medical comorbidities. The most common comorbidities were: hypertension (35%), diabetes (13%), and heart disease (10%). Table 3 presents the results of the analyses for demographics, clinical characteristics, and medical comorbidities while controlling for age, EOR, and tumor location. Prior history of asthma (hazard ratio [HR]: 2.63; 95% confidence interval [CI]: 1.24–5.58; p = 0.01), hypercholesterolemia (HR: 1.95; 95% CI: 1.09–3.50; p = 0.02), and urinary incontinence (HR: 2.29; 95% CI: 0.95–5.57; p = 0.07) were associated with shorter overall survival (p < 0.15), while controlling for age, tumor location, and EOR.

**Table 2:** Pre-existing medical comorbidities of patients in sample.

Medical Comorbidity	Frequency (%)
Arthritis	9 (6%)
Asthma	9 (6%)
Previous cancer	15 (9%)
Depression/Anxiety	16 (10%)
Diabetes	21 (13%)
Heart disease	17 (10%)
High blood pressure	57 (35%)
Hypercholesterolemia	16 (10%)
Incontinence	6 (4%)
Migraines	8 (5%)
Seizures	7 (4%)
Stomach ulcer/upset	9 (6%)
Stroke	6 (4%)
Thyroid problems	8 (5%)

**Table 3:** Hazard ratios and confidence interval of individual-characteristic analyses adjusted for known cofounders.

Characteristic	Hazard ratio	95% CI	p-value
Sex (ref = male)	1.28	0.91, 1.79	0.16
Race (ref = nonwhite)	1.15	0.77, 1.72	0.48
Insurance (ref = uninsured)			0.35
*Commercial*	0.61	0.29, 1.29	
*Government*	0.79	0.38, 1.64	
Married	0.87	0.60, 1.25	0.44
Alcohol use	0.87	0.59, 1.28	0.48
Tobacco use (ref = never)			0.42
*Current*	1.37	0.65, 2.89	
*Former*	1.57	0.76, 3.21	
Arthritis	0.84	0.41, 1.72	0.63
**Asthma**	**2**.**63**	**1**.**24**, **5**.**58**	**0**.**01**
Previous cancer	1.36	0.77, 2.41	0.29
Depression or anxiety	1.39	0.81, 2.40	0.23
Diabetes	1.04	0.63, 1.71	0.88
Heart Disease	1.12	0.64, 1.95	0.70
High blood pressure	1.17	0.82, 1.68	0.39
**Hypercholesterolemia**	**1**.**95**	**1**.**09**, **3**.**50**	**0**.**02**
**Incontinence**	**2**.**29**	**0**.**95**, **5**.**57**	**0**.**07**
Migraines	1.19	0.54, 2.62	0.66
Seizures	0.57	0.21, 1.55	0.27
Stomach ulcer/upset	1.01	0.47, 2.17	0.98
Stroke	1.71	0.71, 4.12	0.23
Thyroid disease	1.75	0.79, 3.88	0.17
Tumor volume	1.00	0.99, 1.01	0.84

All analyses controlled for age, EOR, and tumor location. CI = Confidence Interval.

The final multivariable model included age, tumor location, EOR, asthma, hypercholesterolemia, and urinary incontinence (Table 4). Multivariable analysis demonstrated that increasing age (HR: 1.02; 95% CI: 1.01–1.04; p < 0.01), asthma (HR: 2.22; 95% CI: 1.02–4.83; p = 0.04), and hypercholesterolemia (HR: 1.99; 95% CI: 1.11–3.56; p = 0.02) were each associated with shorter overall survival. Urinary incontinence was not associated with survival (HR: 1.96; 95% CI: 0.78–4.92; p = 0.15) in the multivariable model. Complete resection (HR: 0.52; 95% CI: 0.34–0.80, p < 0.01) and near total resection (HR: 0.58; 95% CI: 0.35–0.94; p = 0.03) were both associated with significantly longer survival time when compared to biopsy alone, while subtotal resection (HR: 0.91; 95% CI: 0.51–1.64; p = 0.76) was not significantly protective. Tumor location was not associated with overall survival (p = 0.16).

**Table 4:** Hazard ratios and confidence intervals of final model adjusted for known cofounders and one another.

Characteristic	Hazard ratio	95% CI	p-value
**Asthma**	**2**.**22**	**1**.**02**, **4**.**83**	**0**.**04**
**Hypercholesterolemia**	**1**.**99**	**1**.**11**, **3**.**56**	**0**.**02**
Incontinence	1.96	0.78, 4.92	0.15
**Age** (**per 1 year increase**)	**1**.**02**	**1**.**01**, **1**.**04**	**<0**.**01**
**Extent of resection** (**ref** = **biopsy only**)			**0**.**01**
*Subtotal resection*	0.91	0.51, 1.64	0.76
***Near total resection***	**0**.**58**	**0**.**35**, **0**.**94**	**0**.**03**
***Complete resection***	**0**.**52**	**0**.**34**, **0**.**80**	**<0**.**01**
Tumor location (ref = frontal)			0.16
*Temporal*	0.93	0.54, 1.63	
*Parietal*	0.59	0.27, 1.28	
*Occipital*	1.06	0.35, 3.23	
*Multiple locations*	1.42	0.87, 2.31	
*Other*	1.25	0.68, 2.31	

CI = Confidence Interval.

## Discussion

Surgical patients with GBM who had a prior history of asthma or hypercholesterolemia had a significantly higher relative risk of mortality in multivariable analysis. Sex, race, and other medical comorbidities were not significantly associated with survival in this study. The poor prognosis of patients with GBM following diagnosis likely negates the hazardous effects of other medical comorbidities on survival. Many of such other diseases require years to cause fatal complications or contribute otherwise to earlier death.

One hypothesis for these findings is that there is a unique interaction between glioblastoma and these comorbidities. The pathophysiology behind asthma and hypercholesterolemia could influence the progression or development of GBM. Prior studies have found that patients with atopic diseases including allergies, eczema, and asthma have a decreased susceptibility to grade II and III gliomas and improved prognosis^26^. It has been shown that there is an inverse relationship between serum IgE levels and risk of glioma, and high amounts of mast cells have been detected in GBM tissues^27,28^. Gohar *et al*.^28^ linked certain IL-4Rα and IL-13 alleles to increased glioma susceptibility, and found the IL-4Rα AA genotype in GBM patients was associated with prolonged survival. A 2011 study by Scheurer *et al*.^29^ found that those with a self-reported history of asthma/allergies and regular anti-inflammatory/NSAID use had a protective association with development of GBM, but there was no significant effect of regular antihistamine use on risk of GBM. A later study by Amirian *et al*.^30^ revealed that regular antihistamine use in patients with self-reported asthma/allergy history was associated with increased risk of glioma, but without any observed effect on survival. Atopy is a disease of the immune system, and the immunologic properties of GBM have been well-described^31^. It is reasonable to suspect that systemic diseases affecting the immunologic and inflammatory responses would have an effect on GBM development and prognosis.

Perhaps those individuals with asthma have lesser risk of developing glioma, but are prone to more aggressive tumors or a worse clinical course when the tumor does emerge, which is plausible given the results of this study. It could also be that individuals with atopic diseases are less likely to develop secondary GBM arising from low-grade glioma, due to the previously-reported decreased susceptibility to lower-grade gliomas in these patients. Since primary GBM has a worse prognosis than secondary GBM this could explain the poorer survival seen in this sample. The relationship between atopy and GBM is actively being researched, and the relationship between asthma and overall survival in particular needs additional evidence.

There is less research on the relationship between hypercholesterolemia and GBM. Studies have shown the importance of cholesterol on GBM cell survival^32^. The effect of statin use on GBM has been better studied with data suggesting statins reduce risk of seizure prior to diagnosis, but do not significantly affect survival following diagnosis^33^. A study by Gaist *et al*.^34^ demonstrated a dose-dependent survival benefit in those taking statins prior to diagnosis of GBM. This suggests that the level of control or treatment of hypercholesterolemia at the time of diagnosis may be an underlying factor explaining our observed relationship between hypercholesterolemia and overall survival.

Another hypothesis to explain the association between asthma and hypercholesterolemia and decreased survival is that these diseases could be fatal or lead to organ failure within a short time frame. Asthma is associated with increased post-operative pulmonary complications, including infection, asthma exacerbation, and acute respiratory failure^35^. Beyond the post-operative period, asthma still remains an immediate threat to life due to pneumonia and/or acute respiratory failure^36^. Hypercholesterolemia was associated with poorer overall survival within this study, but an explanation for these findings remains unclear. Hypercholesterolemia is related to myocardial infarction and other acute vascular events that can be rapidly fatal especially within the acute post-operative period. It would be expected that a history of heart disease and stroke would exhibit a similar relationship as hypercholesterolemia with overall survival, which was not observed within this study.

Prior studies evaluating comorbidities and long-term survival in patients with GBM have focused on a limited number of comorbid conditions, primarily diabetes mellitus^19,37^. Additionally, the usual risk of mortality associated with hypertension, diabetes, stroke, and heart disease may be overcome by the drastically higher rate of mortality of patients within our sample population. It could be that individuals in our study with these diagnoses, which would reasonably be expected to cause many of the same acute vascular events as hypercholesterolemia, were already well-treated following prior diagnoses and thus did not experience these complications after their diagnosis of GBM. The other comorbidities measured in our sample, such as arthritis, incontinence, seizures, migraines, depression/anxiety, and thyroid disease, may increase the comorbid burden and poorer overall health status of patients, but would not be expected to increase the risk of mortality within the shortened life expectancy of patients presenting with GBM.

In our study, the observed probability of survival at 6-, 12-, 18-, and 24-months was 71%, 50%, 34%, and 26%, respectively, which is consistent with estimates of median survival from previous studies^2,3^. There was a clear male predominance amongst patients who presented to our institution with GBM, which has been demonstrated in nationwide samples^2,7^. In controlling for surgical EOR and age, we saw that complete resection and near total resection both were associated with significantly longer survival time when compared to biopsy alone, while subtotal resection was not significantly protective. This finding is consistent with previous studies suggesting the survival benefit of complete resection^7,18^. Increasing age was associated with decreased survival, which has been repeatedly demonstrated in the literature^9,10^. Tumor location was not associated with overall survival. Prior research has demonstrated that tumor location influences overall survival via surgical approaches, oncological management, and the ability to pursue surgical resection^25^. Our findings could be explained by not stratifying our tumor locations based on eloquence, or by preselecting surgical candidates based on those whose tumors were more amenable to surgery and underwent resection rather than conservative management alone.

### Limitations

Limitations of this study include its retrospective nature and power. The sample size is comparable to other single-institution studies, although there was a limited sample size for subgroups for some comorbidities^5,19^. This sample has a higher proportion of nonwhite patients compared to other studies on glioblastoma patients in the literature^2^. This may account for some of the divergence in findings and also may improve the applicability of these findings to medical centers with diverse patient populations. We did not evaluate tumor biomarkers, such as MGMT, IDH1/2, P53, EGFR, and PTEN, that are commonly related to overall survival, as these were not consistently available at the time of presentation to our institution for this study sample (2005–2015). VCU is an academic medical center with a large referral base, thus patients in the sample may have been diagnosed with a brain mass at an outside facility before coming to VCU for treatment. In addition, the 9% of patients who were censored at the date of their last clinic visit could have survived for significant amount of time afterwards, which would not be captured in this dataset. Lastly, we did not assess the underlying cause of death for each patient, which could further delineate whether progression of disease or other medical comorbidities were the attributable factor for mortality within this patient population.

### Future directions

Future research with larger sample sizes using prospective or multi-institutional design would be valuable for investigating comorbid predictors of survival in GBM. Specifically, the underlying pathophysiology for asthma or hypercholesterolemia in relation to GBM should be investigated to understand how each disease process alters the overall length of survival. Properly treated hypercholesterolemia or asthma could potentially alter prognoses when compared to untreated disease in this population. The effects of lipid-lowering and asthma/allergy medications on the natural history, progression, and survival of patients with GBM is one possible avenue of further investigation. An examination of tumor markers present in patients with history of asthma or hypercholesterolemia could be useful in assessing potential mechanisms for differences in prognosis of these patients.

## Conclusions

GBM is an aggressive and prevalent primary CNS tumor with unfavorable overall survival and few known prognostic factors. This study demonstrates that, in addition to patient age and decreased extent of resection, history of asthma and hypercholesterolemia are each associated with a worse prognosis in GBM. Further research should establish the interactions of the pathophysiology of these diseases with glioblastoma and whether or not optimal medical control of asthma and hypercholesterolemia provides survival benefit. Recognizing and ensuring that comorbid conditions are adequately treated remains essential in the treatment of patients with GBM.

## Data Availability

The datasets generated during and/or analyzed during the current study are available from the corresponding author on reasonable request.
